# Recombinant gonadotropin therapy to improve spermatogenesis in nonobstructive azoospermic patients – A proof of concept study

**DOI:** 10.1590/S1677-5538.IBJU.2022.99.13

**Published:** 2022-02-10

**Authors:** Rita Jacubcionyte Laursen, Birgit Alsbjerg, Helle Olesen Elbaek, Betina Boel Povlsen, Kirsten Brock Spanggaard Jensen, Jette Lykkegaard, Sandro C. Esteves, Peter Humaidan

**Affiliations:** 1 Skive Regional Hospital The Fertility Clinic Skive Denmark The Fertility Clinic, Skive Regional Hospital, Skive, Denmark; 2 Universidade Estadual de Campinas Divisão de Urologia Departamento de Cirurgia Campinas SP Brasil Departamento de Cirurgia, Divisão de Urologia, Universidade Estadual de Campinas (UNICAMP), Campinas, SP, Brasil; 3 Clínica de Andrologia e Reprodução Humana Centro de Referência em Reprodução Masculina Campinas SP Brasil ANDROFERT, Clínica de Andrologia e Reprodução Humana, Centro de Referência em Reprodução Masculina, Campinas, SP, Brasil; 4 Aarhus University Faculty of Health Aarhus Denmark Faculty of Health, Aarhus University, Aarhus, Denmark

**Keywords:** Azoospermia, Nonobstructive [Supplementary Concept], Infertility, Male, Spermatozoa

## Abstract

**Purpose:**

Nonobstructive azoospermia (NOA) associated with primary spermatogenic failure is a common cause of male infertility usually considered untreatable; however, some reports have suggested that hormonal stimulation to boost the intra-testicular testosterone level and spermatogenesis might increase the chance of achieving pregnancy using homologous sperm.

**Materials and Methods:**

We report a series of eight NOA males who received long-term treatment with recombinant human chorionic gonadotropin twice a week for spermatogenesis stimulation. Six males received additional recombinant follicle-stimulating hormone (FSH) supplementation 150-225 IU twice weekly.

**Results:**

After recombinant gonadotropin therapy, viable spermatozoa were retrieved from the ejaculate in two patients and by testicular sperm aspiration (TESA) in another two subjects. Singleton spermatozoon retrieved from testes were frozen by vitrification on Cell-Sleeper devices. Two live births were obtained after intracytoplasmic sperm injection with ejaculated spermatozoa and one live birth and an ongoing pregnancy using thawed spermatozoa from TESA.

**Conclusion:**

Our proof-of-concept study indicates that hormonal therapy with recombinant gonadotropins could be considered in infertile men with NOA as an alternative to sperm donation. Large-scale studies are needed to substantiate hormone stimulation therapy with recombinant gonadotropins in routine clinical practice for this severe form of male infertility.

## INTRODUCTION

Azoospermia is defined as the absence of spermatozoa in the ejaculate following centrifugation (1). It is a common reason for infertility, affecting about 10-15% of infertile males (1, 2). Approximately 60% of all azoospermia cases are related to impaired spermatogenesis, known as nonobstructive azoospermia (NOA). In contrast, in ∼40% of cases, azoospermia is caused by blockage of the ductal system (i.e., obstructive azoospermia) (2, 3). NOA is challenging for couples seeking parenthood who wish to use their own gametes, especially in countries with strong cultural and religious beliefs (4). Notably, the absence of sperm in the ejaculate does not always reflect a complete lack of spermatogenesis because, in some males, it is possible to identify residual sperm production in the testicular tissue (2, 5). The most effective surgical technique to retrieve sperm from these focal areas is microdissection testicular sperm extraction (micro-TESE), after which they can be used either immediately for intracytoplasmic sperm injection (ICSI) or cryopreserved for future use (2, 5, 6).

Non-obstructive azoospermia is typically considered untreatable; however, recent reports have shown that sperm maturation might be boosted by selective estrogen-receptor modulators and exogenous urinary gonadotropins (5, 7–11). The reasons why some individuals respond to treatment whereas others do not are largely unknown but might be related to the fact that in some patients, spermatogenesis is arrested during the early or later stages. Yet, until now, there is minimal evidence concerning the effectiveness of gonadotropin therapy for males with NOA.

The use of recombinant gonadotropins in preference over urinary counterparts might be advantageous due to their increased efficacy, safety, and patient-centeredness profile (12). However, only two case reports explored the combined use of recombinant human chorionic gonadotropin (hCG) and recombinant follicle-stimulating hormone (FSH) to treat this condition (10, 11). Importantly, the knowledge about side effects, risks, and reproductive outcomes after hormonal stimulation with urinary or recombinant gonadotropins in NOA males is scanty.

The primary aim of this study was to evaluate the role of recombinant gonadotropin therapy for spermatogenesis stimulation in patients with NOA due to spermatogenic failure. Secondary aims were to report the clinical outcomes of treatment and possible side effects.

## MATERIALS AND METHODS

A total of eight patients with NOA admitted to a public fertility clinic in Denmark received gonadotropin therapy with recombinant drugs for spermatogenesis stimulation since December 2016. Before treatment, all patients had a diagnostic testicular sperm aspiration (TESA) showing either no sperm or only few non-viable sperm. Patients were informed that recombinant gonadotropin therapy for their condition was off-label and that evidence concerning its effectiveness and safety was minimal.

The standard evaluation included medical history, physical examination, repeated semen analyses with the examination of pelleted semen, ultrasound of testes, basic hormone evaluation, and genetic studies, as previously described (10). In all patients, NOA was confirmed by testicular histopathology of specimens taken by TESA. Most patients had an unremarkable history explaining NOA, although one patient reported cryptorchidism. Notably, one patient reported a history of long-term anabolic steroid use. Although such a case would best fit in the category of NOA due to hypogonadotropic hypogonadism (10), this particular patient had an atypical clinical presentation. Specifically, baseline endogenous FSH and LH levels were markedly elevated, and azoospermia was found in repeated semen analyses even though the patient had stopped using steroids for several years (Table-1). Another patient had a history of anejaculation due to spinal cord injury, and again, an atypical presentation as azoospermia was noticed on examination of specimens obtained by electroejaculation.

**Table 1:** Baseline patient characteristics.

Case No.	Age (years)	Medical history	Testis size (mL) L/R	Baseline hormonal serum levels				Testicular histopathology
				FSH IU/l	LH IU/l	E2 pmol/l	T nmol/l	
1	28	Cryptorchidism	8/8	11.6	8.0	111.0	18.2	Hypospermatogenesis 2
2	30	Unremarkable	10/8	8.3	6.4	82.8	14.1	Early maturation arrest 1
3	38	Left inguinal hernia repair	8/12	37.0	11.9	<18.0	9.2	Early maturation arrest^1^
4	45	Unremarkable	10/10	15.1	6.5	44.7	6.2	Sertoli cell only 1
5	33	Unremarkable	12/12	48.0	14.9	<18.0	11.5	Early maturation arrest 1
6	34	Long-term anabolic steroid abuse	15/15	34.0	14.0	30.0	11.5	Late maturation arrest 1
7	40	Unremarkable	4/4	8.3	9.4	23.5	3.1	Hypospermatogenesis 2
8	42	Tetraplegia; anejaculation *	10/10	7.5	2.7	31.9	6.3	Hypospermatogenesis 2

1 No sperm observed on diagnostic testicular sperm aspiration

2 Rare nonmotile and morphologically abnormal sperm observed on diagnostic testicular sperm aspiration

* Azoospermia on examination of specimen obtained by electroejaculation

**L** = left; R = right; **IU** = International units

Three cases were histologically diagnosed as early maturation arrest, three cases as hypospermatogenesis, one case as a late maturation arrest, and one case as Sertoli-cell only (Table-1). All patients had a normal karyotype, no Y chromosome microdeletions, or cystic fibrosis pathogenic variants. Furthermore, none of the males had clinically significant varicoceles.

### Hormonal Treatment

After signed informed consent, the patients started treatment with human chorionic gonadotropin (rec-hCG; choriogonadotropin alfa, Ovitrelle 250 micrograms/0.5 mL prefilled pen ready for injection, Merck) 60 mcg, subcutaneously, twice weekly. The 60-mcg dose corresponds to approximately 1620 IU of the drug, and the medication was self-administered using a pen device. A doctor or a nurse taught patients how to set the dose and administer the medication. To set the amount, patients were instructed to gently rotate the setting knob of the pen device clockwise until six audible ‘clicks’ were reached. We estimated that each audible click corresponded to approximately ten micrograms (mcg) of rec-hCG (∼270 IU), considering that the pen contains 250 mcg of rec-hCG and the setting knob has 25 audible clicks when rotated until the end. Notably, information about the bioequivalence of the fractioned dose was not available in the technical or patient user’s manual as the pen is intended for single-use in women undergoing infertility treatment.

**Figure 1:**
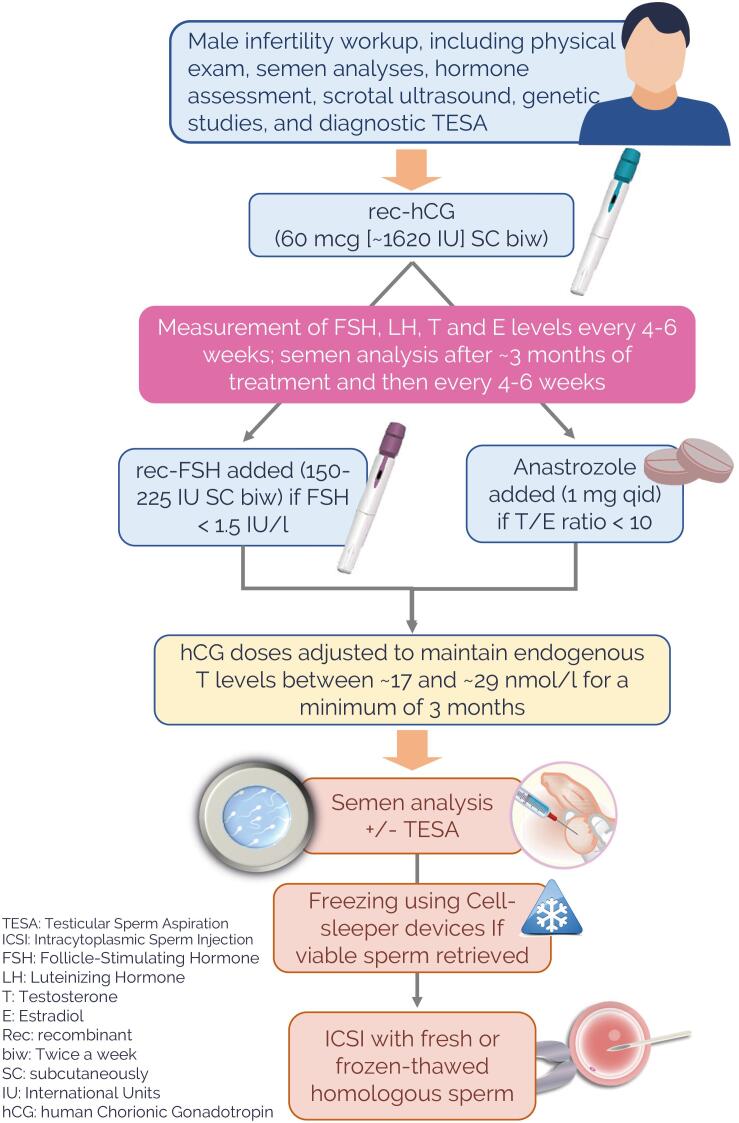
Illustration depicting the hormonal treatment algorithm.

Six patients received additional subcutaneous injections of recombinant follicle-stimulating hormone (rec-FSH; follitropin alfa, Gonal-f 300 IU/0.5 mL, prefilled multidose pen ready for injection, Merck) 150-225 IU, subcutaneously, twice weekly. Figure-1 depicts the treatment algorithm, and Table-1 details the characteristics of the treated patients. The mean baseline FSH level was 21.2 IU/l (range 7.5-48), and the serum testosterone (T) level was 10 IU/l (range 3.1-18.2) before initiation of treatment.

During treatment, semen analyses and hormone testing were performed periodically. Specifically, serum FSH, luteinizing hormone (LH), estradiol, and T levels were monitored every three to four weeks. Notably, the blood sampling was standardized to 48-50 hours after the last hCG injection and before 10 a.m. to minimize the influence of circadian variation (13). Semen analysis was carried out three months after treatment commencement and then periodically every four to 6 weeks.

The rec-hCG dosing was adjusted to maintain the T level between 17 and 29 nmol/l, and when and if the serum FSH level dropped below 1.5 IU/l, supplementation with rec-FSH (150-225 IU) twice weekly commenced and continued for at least three months. Moreover, if the T to estradiol ratio turned <10 (ng/dL:pg/mL), an aromatase inhibitor (anastrozole, 1mg daily) was added and continued as needed. The computation above was made after converting T and estradiol levels as follows: Testosterone: 1 ng/dL = 28.818 nmol/L; Estradiol: pg/mL = 0.272 pmol/L.

## RESULTS

The mean age of the males was 36.3 years. All patients received long-term treatment with rec-hCG for spermatogenesis stimulation for a mean of 10 months (range: 8 to 15 months). After recombinant gonadotropin therapy, viable spermatozoa were seen in the ejaculate in two cases and retrieved by TESA in another two cases (Table-2). The Cell-Sleeper method, previously described by Endo et al. and Coetzee et al. (14, 15), was used for sperm cryopreservation. In four patients, spermatozoa were neither found on the examination of pelleted semen nor obtained by TESA after treatment.

**Table 2:** Treatment characteristics and outcomes.

Case No.	Treatment regimen			Treatment duration (months)	Sperm retrieved post-treatment	Sperm freezing	Pregnancy by ICSI using homologous sperm
	hCG dose (IU) (initial/final)	FSH dose (IU) (initial/final)	IA added				
1	1620/1080	150/225	No	9	Ejaculate	Yes	Yes; Live birth
2	1620/1080	150/150	No	8	No	–	–
3	1620/1080	150/150	No	10	No	–	–
4	1620/2700	150/150	Yes	10	No	–	–
5	1620/1620	150/150	No	9	No	–	–
6	1620/810	NA	No	8	Ejaculate	Yes	Yes; Live birth
7	1620/3240	NA	No	14	TESA	Yes	Yes; Live birth
8	1620/1080	150/200	No	15	TESA	Yes	Yes; Ongoing

**TESA** = Testicular sperm aspiration; **ICSI** = Intracytoplasmic sperm injection; **IU** = International units; **hCG** = human chorionic gonadotropin; **FSH** = Follicle-stimulating hormone; **AI** = Aromatase inhibitor; **NA** = not applicable

Hormonal treatment with recombinant gonadotropins resulted in two live births after ICSI with ejaculated spermatozoa and one live birth using thawed spermatozoa from TESA. The last couple (patient No. 8) achieved a clinical pregnancy after ICSI using frozen-thawed sperm retrieved by TESA, which is ongoing.

No adverse effects were recorded during the treatment period. However, patient No. 4 exhibited elevated estradiol levels during treatment and required the addition of an aromatase inhibitor to keep the testosterone to estradiol ratio within optimal levels. In this patient, anastrozole was used for nine months. In case No.5, a right-sided testicular seminoma was diagnosed after nine months of treatment. The patient underwent unilateral orchiectomy, and the couple subsequently obtained a pregnancy and live birth after in vitro fertilization using donor sperm.

## DISCUSSION

We herein presented a proof-of-concept study based on a case series of eight males with NOA and failed TESA treated with recombinant gonadotropin therapy for spermatogenesis stimulation. After treatment, viable sperm were obtained in four cases, resulting in three healthy children and one ongoing pregnancy by ICSI.

### Stimulation of spermatogenesis with exogenous gonadotropins

Outside the context of NOA, it has been suggested that 58-80% of men with idiopathic infertility might be sensitive to exogenous FSH stimulation since these patients have FSH receptor gene polymorphisms (16, 17) and, thus, could benefit from an increase in their circulating FSH level. It has been estimated that a total of 10-18 males with idiopathic infertility need to undergo medical treatment with exogenous gonadotropins to achieve one additional pregnancy (18). As gonadotropin therapy is costly and the evidence concerning its effectiveness is limited, it is debated whether FSH as a standard treatment for males should be recommended (19). Despite that, recently, gonadotropin therapy was approved to treat idiopathic male infertility in Italy for patients with a serum FSH level of less than 8 IU/L. Interestingly, Santi et al. reported an increase in sperm concentration and morphology in about half of the treated males and, importantly, without any adverse events during treatment (20). Furthermore, two small meta-analyses evaluating natural conception rates after gonadotropin therapy reported an odds ratio (OR) of 4.94 (CI:2.13-11.44) and 4.5 (CI:2.17-9.33) in favor of treatment (18, 21). Despite these promising results in men with idiopathic infertility, there is no consensus on using gonadotropins in NOA males with spermatogenic failure (22), and treatment is not routinely recommended (23, 24).

Moreover, data about the clinical utility of recombinant gonadotropins in the context of NOA is minimal. To our knowledge, only two case reports by our group have been published (10, 11). In one report, a full-term delivery of a healthy child was obtained with the aid of ICSI using ejaculated sperm in an infertile couple whose male partner had NOA due to cryptorchidism (patient No. 1 in the current series) (10). The patient received long-term hormonal stimulation with combined use of rec-hCG and rec-FSH, cryopreservation of ejaculated spermatozoa using the cell sleeper method, and subsequently ICSI. In another report, two NOA patients with testis biopsy revealing maturation arrest were treated similarly (11). In one case, the patient remained azoospermic. A micro-TESE, carried out six months after treatment, successfully harvested sperm; the couple achieved a live birth delivery by ICSI using testicular sperm. The second case was more challenging, as only morphologically abnormal (mainly globozoospermic sperm) were retrieved by micro-TESE after one year of therapy. Despite two ICSI trials and transfer of apparently healthy embryos to the uterine cavity, the couple remained childless as no implantation occurred.

### Clinical Interpretation

A crucial step in the sperm maturation process is maintaining a physiological intra-testicular T level (ITT), which is 50-100-fold higher than the levels in circulation (25). HCG stimulates LH-receptors on Leydig cells resulting in an increased T production, which in synergy with Sertoli cell stimulation by FSH further promotes spermatogenesis (2, 7–9). Indeed, a positive relationship exists between a normal serum T level (vs. a low T level) and a higher chance of sperm retrieval (OR 1.63, 95% CI 1.08-2.45, p=0.02) (24). However, no data support a clear threshold of serum T levels facilitating optimal spermatogenesis.

In humans, spermatogenesis is stimulated by FSH and T in synergy; FSH mainly stimulates the first stages of spermatogenesis (FSH-dependent phase), including determination of Sertoli cell number, spermatogonial proliferation, stimulation of meiotic progression until spermatid stage, and transport of nutritive substances to germ cells. In contrast, T supports the post-meiotic advancement of round spermatids to mature sperm (testosterone dependent phase) (19). The combined action of the two gonadotropins in cases of NOA associated with hypergonadotropic hypogonadism could either induce or increase spermatogenesis and thus, enhance the chances of obtaining sperm for biological offspring through ART.

It is generally believed that empirical hormonal therapy with FSH for NOA males with primary testicular failure is ineffective because baseline serum gonadotropin levels are already elevated. However, it has been shown that excessive circulating FSH levels might induce FSH receptors down-regulation on the Sertoli cell (26–30). Interestingly, hCG treatment decreases circulating FSH levels (7, 10, 11), which are typically elevated in most NOA patients. Thus, an FSH reset to normal levels might reduce Sertoli cell desensitization caused by excessive circulating FSH and enhance Sertoli cell function (31, 32). However, in some NOA men treated with hCG, FSH levels are profoundly suppressed (e.g., below 1.5 IU/L) (7, 10, 11). These patients need exogenous FSH supplementation to optimally stimulate the Sertoli cells and spermatogonia proliferation (2, 7–11, 31).

The mean duration of therapy for the present case series was ten months. In contrast, most studies, including those using gonadotropin therapy for idiopathic male infertility, reported outcomes after only three months of treatment (8–10, 20, 33). Our case series focused on achieving optimal T and FSH levels before starting fertility treatment, and therapy continued during the subsequent IVF treatment. If a pregnancy was achieved, treatment continued until at least the seventh week of pregnancy.

### Sperm Retrieval

There is no agreement on whether a diagnostic testicular biopsy should be performed before stimulation with exogenous gonadotropins. NOA patients with hypergonadotropic hypogonadism have an increased number of interstitial testicular lesions (containing no Leydig cells) and fibrosis compared with obstructive azoospermia patients (32). Interestingly, Oka et al. (32) observed an association between hCG use, decreased interstitial lesions, and Leydig cell hypertrophy, which might increase the sperm retrieval rate.

As regards the retrieval method, higher sperm retrieval rates have been reported using micro-TESE than TESA or conventional TESE in the general NOA population (2, 6, 34–37). However, data on sperm retrieval after gonadotropin therapy is minimal. In two studies by the same authors, a 10-21% sperm retrieval rate by micro-TESE was reported in a group of males stimulated with gonadotropins after failed sperm retrieval (7, 38). Yet, micro-TESE is an advanced procedure, requiring a fully equipped operating theatre and a trained microsurgeon, which is unavailable in our Unit.

Collectively, we were able to harvest sperm from 50% of patients, considering both TESA and ejaculated specimens. Although our results are higher than that reported in the literature, it should be noted that our population comprises patients with a good prognosis for sperm retrieval. In our series, a single patient had Sertoli-cell only on testis biopsy, whereas the remaining patients had either maturation arrest or hypospermatogenesis. Previously, only NOA males with late maturation arrest and hypospermatogenesis have been shown to benefit from gonadotropin therapy in terms of the highest chance of sperm retrieval (39), which is in line with our data. Hypospermatogenesis and maturation arrest are typical findings in NOA males. In a 2021 study including 918 patients with NOA, testis biopsy specimens taken during micro-TESE showed hypospermatogenesis (either pure or mixed) in 16.6% and maturation arrest (either pure or mixed) in 30.6% of individuals (40).

In the present study, all couples whose partners had sperm harvested succeeded in achieving a pregnancy. Despite favorable, this finding should not be perceived as indicative of a high pregnancy rate in NOA cases. In a recent systematic review of studies evaluating pregnancy outcomes by ICSI using testicular sperm retrieved from NOA males by micro-TESE, the pooled clinical pregnancy rate was 39% (range 12.2–72.4%), and live birth delivery was achieved in 24% of couples (range 11.8–62.1%) (35). Interestingly, in our series, the pregnancies were achieved in subjects with late maturation arrest and hypospermatogenesis but not in cases with early maturation arrest. However, due to a limited number of patients included, no conclusive recommendation can be made on the ideal candidates for treatment.

### Sperm Cryopreservation

Freezing of few spermatozoa is a time-consuming procedure that requires skilled laboratory staff. In the present series, the Cell-Sleeper method was used. This technique was initially described by Endo et al. (14) and Coetzee et al. (15), who reported a sperm recovery rate of 83% and 88%, respectively, which is in line with our experience.

Sperm cryopreservation after gonadotropin therapy is highly recommended as it will allow repeat ICSI cycles, thus enhancing the likelihood of achieving biological parenthood. The method used in our study allows the preservation of 10-15 spermatozoa per device, which optimized the sperm usage for ICSI as in most cases, the number of oocytes collected does not surpass that number.

### Adverse Effects of Gonadotropins Therapy

In general, gonadotropins are considered safe for stimulating spermatogenesis in the context of idiopathic infertility and hypogonadotropic hypogonadism (17, 19, 33, 41). Only a few side effects like gynecomastia and temporary mastalgia were reported in patients with excessive endogenous T and estradiol levels (33). In our series, no such adverse effects were reported. However, one patient was diagnosed with testicular cancer after nine months of therapy. We speculate that the diagnosis of testicular cancer was likely coincidental as it is well established that NOA patients have an increased risk of developing testicular cancer (hazard ratio: 3.3, 95% confidence interval [CI] 1.6–6.9) (42, 43).

Nevertheless, a previous case report described the development of a seminoma after prolonged FSH and hCG therapy (44). This report concerned a 43-year-old patient with oligozoospermia, normal serum gonadotropins, and an unremarkable ultrasound testicular examination, treated with exogenous FSH for one year. The patient presented with bilateral testicular tumors two years later, and histology revealed a bilateral seminoma.

Although it is currently unknown if gonadotropin therapy might stimulate a pre-existing carcinoma in situ to develop into a testicular tumor, considering the above, our recommendation is to perform repeated testicular ultrasound scans: before initiating therapy, six months later, and at the end of treatment. Moreover, the patient should be encouraged to perform testicular self-examination and contact the handling clinic if any abnormality is detected during or after treatment.

### Patient Counselling

Currently, it has still to be determined which patients could benefit from gonadotropin therapy in a clinical setting. Due to the high cost and long duration of treatment and the uncertainties of sperm acquisition, it is critical to thoroughly discuss the pros and cons of hormonal therapy. Notably, an evaluation of the chance of obtaining a pregnancy should be considered, accounting for the female partner’s age. A diagnostic testicular biopsy providing the dominant histological pattern of the testes before initiating therapy could help in patient selection.

The chances of achieving a pregnancy through sperm retrieval and ICSI without gonadotropin therapy need to be weighed against the cost of treatment and potential side effects. Moreover, introducing this new male treatment regimen raises essential questions about the most optimal treatment plan, sperm retrieval method, and cryopreservation technique when a minimal number of spermatozoa are present. Lastly, the monitoring process during treatment, possible side effects, and the future need for exogenous T supplementation in low-level T males after sperm retrieval must be considered.

Notwithstanding the above considerations, recombinant technology has fulfilled the need for a more reliable source of FSH and hCG, particularly in female infertility treatment (12, 45). While urine-derived gonadotropins require large amounts of human urine as a primary source for manufacturing, the production and purification of recombinant gonadotropins are subjected to continuous quality control assessments, ensuring a pure, consistent, and high-quality product (45). The manufacturing process typically utilizes genes coding for the human FSH and hCG, which are incorporated into the nuclear DNA of a host cell via a plasmid vector, using spliced DNA strings containing the gonadotropin gene and segments of bacterial DNA. Recombinant gonadotropins are generally presented as ready-to-use solutions filled in pen devices, increasing treatment compliance as the patient can inject the drug subcutaneously (12, 45).

### Strengths and Limitations

Our study has several limitations related to the nature of a proof-of-concept study based on a small case series. Despite that, to our knowledge, this is the first series reporting the combined use of recombinant gonadotropins to stimulate spermatogenesis in men affected by NOA. To enhance the appeal of our article, we discussed the current evidence associated with the use of gonadotropin therapy in men affected by this severe form of male infertility. Our literature review only found studies using urinary gonadotropins. Therefore, our study adds to the current literature as recombinant drugs have potential advantages over urinary products.

Another limitation relates to the heterogeneous characteristics of treated patients. Of note, two patients (No. 6 and No. 8) do not perfectly fit in the NOA-spermatogenic failure classification. Patient No. 6, despite reporting a history of anabolic steroid abuse, remained azoospermic even after stopping the drugs for several years. Notably, his baseline endogenous FSH and LH levels were found to be remarkably elevated, thus not fulfilling the classic hypogonadotropic hypogonadism profile that would be expected in such a case. We speculate that this patient had some degree of primary testicular impairment unrelated to the use of anabolic steroids, exacerbated after its prolonged use. Patient No. 8 was primarily characterized as having anejaculation due to spinal cord injury. Still, he produced azoospermic ejaculates after electroejaculation, his testes were hypotrophic, and the hormone profile suggested a primary testicular deficiency. Notwithstanding these observations, all patients fulfilled the criteria of NOA due to primary testicular deficiency based on the clinical picture and unequivocal testis histopathology findings. Importantly, none of them fulfilled the criteria of NOA due to hypogonadotropic hypogonadism, as their baseline hormone levels were either markedly elevated or within normal ranges.

## CONCLUSIONS

Recombinant gonadotropin therapy for males with NOA and spermatogenic failure seems to be a valuable strategy to overcome infertility. In our case series of eight patients, hormonal treatment provided satisfactory results overall with no apparent side effects. It resulted in the retrieval of viable spermatozoa for ICSI in four cases, resulting in three live births and one ongoing pregnancy. Our findings suggest that hormonal stimulation with recombinant gonadotropins could be considered for selected infertile men with NOA as an alternative to sperm donation. Nevertheless, the evidence supporting its use remains minimal and, therefore, large-scale studies are warranted to determine its efficacy and safety.
